# Anti-nuclear antibodies in patients with suspicion of neuromyelitis optica spectrum disorder: Frequency and significance

**DOI:** 10.12669/pjms.42.6.15417

**Published:** 2026-06

**Authors:** Farva Jalil, Shafain Sheikh, Arsalan Ahmad, Tahir Aziz Ahmed

**Affiliations:** 1 Farva Jalil, *MBBS, Post Graduate Resident (Immunology)*, Department of Immunology, Shifa International Hospital, Islamabad, Pakistan; 2 Shafain Sheikh, *MBBS, FCPS (Immunology)*, Department of Immunology, Shifa International Hospital, Islamabad, Pakistan; 3 Arsalan Ahmad, *MBBS, MD (Neurology)*, Department of Neurology Shifa International Hospital, Islamabad, Pakistan; 4 Tahir Aziz Ahmed, *MBBS, FRCPath Immunology (UK)*, Department of Immunology, Shifa International Hospital, Islamabad, Pakistan

**Keywords:** Anti Aquaporin-4 antibody, Anti-nuclear antibodies, Autoimmune comorbidities, Neuromyelitis optica spectrum disorder

## Abstract

**Backgroud & Objectives:**

Neuromyelitis Optica spectrum disorder (NMOSD) is an autoimmune inflammatory disease involving optic nerves and spinal cord. The association of NMOSD with anti-nuclear antibodies and autoimmune disorders has been reported. This study was conducted to determine frequency of anti-nuclear antibodies (ANA) in patients with NMOSD and to observe for the development of symptoms related to autoimmune disorders on follow up.

**Methodology:**

After approval from Institutional Review Board, this cross-sectional study was conducted at Immunology Department, Shifa International Hospital, Islamabad, Pakistan and after informed verbal consent from patients, serum samples were tested by indirect immunofluorescence for anti-aquaporin four antibodies (AQP-4) and ANA. Clinical data of patient was collected via a structured questionnaire. Follow-up of 6-12 months was conducted in patients who were positive for ANA.

**Results:**

Total 248 patients were included in this study of which 32 were positive for AQP4- Antibodies (mean age 32.4 ± 14.1 years), out of which ANA was positive in 17 (53%) patients whereas in AQP-4 negative group, ANA was positive in 70 (32%) patients. At one year follow-up, in AQP-4 and ANA positive group, two patients were diagnosed with autoimmune thyroid disorders and one patient was diagnosed with Systemic Lupus Erythematosus.

**Conclusion:**

In this study, we concluded that ANA were observed more frequently with AQP-4 antibody positivity. Considering this, detection of ANA in AQP-4 antibody positive patients may help in establishing early diagnosis of an accompanying autoimmune disease leading to early diagnosis and an overall favorable outcome in patients.

## INTRODUCTION

Neuromyelitis Optica spectrum disorder (NMOSD), previously known as Devic’s syndrome is an autoimmune inflammatory disorder involving optic nerves and spinal cord. The prevalence of NMOSD is reported to be approximately 0.3–4.4 per 100,000 with a female preponderance.^1^ NMO was considered a subtype of Multiple sclerosis for several years but the discovery of NMO specific serum autoantibodies in 2006, collectively known as NMO-IgG, helps to distinguish NMO from MS. These NMO-IgG are directed against Aquaporin-4, a water channel protein widely expressed in the brain, spinal cord, and optic nerve, particularly in the foot processes of astrocytes at the blood brain barrier.^2^ NMOSD predominantly effects optic nerves and spinal cord and clinical presentation ranges from mild sensory disturbances to complete transverse myelitis with tetraplegia or paraplegia and bladder–bowel dysfunction.^3^ The association of NMOSD with other systemic autoimmune disorders and autoantibodies has been reported. Clinically, a considerable number of NMOSD patients are positive for other autoimmune antibodies including antinuclear antibodies (ANA) which are also seen in several autoimmune connective tissue diseases including systemic lupus erythematosus, Sjogren syndrome, Rheumatoid arthritis, and systemic sclerosis.^4^ Considering a paucity of published data focusing on relationship between NMOSD with other autoantibodies and autoimmune diseases in local population, this study was conducted aiming to determine the frequency of this association to postulate significance of carrying out ANA testing in NMOSD patients for a favorable outcome. To the best of our knowledge, this Is the first study evaluating relation of NMOSD and anti-nuclear antibodies from Pakistan.

## METHODOLOGY

This cross-sectional study was conducted at Immunology Department, Shifa International Hospital, Islamabad, Pakistan;

### Ethical Approval:

It was obtained from IRB (Institutional Review Board Approval # 232-24; dated June 8, 2024) of Shifa International Hospital.

Sample size was calculated using WHO sample size calculator (CI: 95%, anticipated population proportion for ANA positivity in AQ4 positive samples: 36%^5^, Precision required: 6%). Total 248 patients were included. Patients of both genders and all age groups whose serum samples were sent for AQP-4 antibody testing were included. Serums samples positive for anti MOG antibodies, those for which no clinical data was available or those who were already diagnosed with an existing autoimmune connective tissue disease were excluded from the study. Informed verbal consent was obtained from all patients and from guardians for patients under 18 years of age. Results were retrieved from the database for patients in whom ANA testing had been performed as part of routine workup. For those without prior testing, ANA analysis was performed on serum samples submitted for anti AQP-4 antibody. Anti-AQP- 4 antibody test was carried out by indirect immunofluorescence using EU-90 cells transfected with AQP-4 antigen (Euroimmun Lübeck, Germany).

Indirect Immunofluorescence test using hep-2 cells as a substrate (Euroimmun Lübeck, Germany) was carried out for detection of ANA, according to manufacturer’s instructions. Briefly, patient’s serum sample at a dilution of 1:100 with PBS were incubated with hep-2 cells. After washing, a second incubation step was carried out using fluorescein isothiocyanate labelled anti-human (goat) IgG and slides were viewed under fluorescence microscope. Different patterns and fluorescence intensity for each sample was noted according to international consensus on ANA Pattern (ICAP). In case of no characteristic fluorescence, sample was considered negative for ANA. Positive and negative control was tested along with patient samples, on each slide as an internal quality control measure.

In order to address selection bias, a consecutive sampling approach was used with clearly defined inclusion and exclusion criteria. ANA results were independently reviewed by an analyst blinded to clinical and anti AQP 4 antibody status of the patient.

Clinical information and treatment modalities used were collected via a structured questionnaire by interviewing patients, their relatives, physicians and by reviewing medical records. Patients who had ANA in serum were followed for a period of six months to one year to see for development of symptoms related to any autoimmune connective tissue disorders or autoimmune organ specific disorder.

### Statistical analysis:

Data was expressed as percentages for categorical variables and Mean ± standard deviation for continuous variables. Characteristics were compared using chi-square test for categorical variables. A p- value of <0.05 was considered statistically significant. All statistical analyses were performed by the Statistical Program for the Social Sciences (SPSS) statistical software (version 23.0).

## RESULTS

A total of 248 patients were included in this study, demographics of which are shown in Table-I. Anti AQP-4 ab was positive in 32 patients. Among these, 17 were also positive for ANA (Table-II). Fine speckled was the most frequent pattern of ANAs observed in both AQP-4 antibody positive and negative group. However, in AQP-4 antibody negative group; nine patients showed centrosome pattern of ANA, and seven patients showed cytoplasmic pattern of antibodies whereas none of the patients in AQP-4 antibody positive group showed these patterns. However, these results were not statistically significant (p value= 0.23)

**Table-I:** Gender and age distribution of total study population.

Characteristics	Overall	Females	Males
Number of study participants	n=248	n= 145 (58.4%)	n = 103 (41.5 %)
Mean Age ± SD	32.4 ± 14.1 (Age range: 2-73)	32.9 ± 13.6	32.4 ± 14.1

**Table-II:** Demographics of total study population according to antibody status with frequency of ANA patterns observed in AQP-ab positive and negative samples.

Parameters	Total Number=n (%)	Mean age ± SD	Gender distribution	Positive ANA	P-Value*	ANA Pattern (n)
Aquaporin-4 antibody Positive	n= 32 (12.9%)	33 ± 15.9 (Age range: 6-68)	Females: n= 28 (87.5%) Males: n= 4 (12.5%)	n=17 (53%) Females: n= 15 (88%) Males: n= 2 (11.7)	0.03	Fine speckled: 13 Nucleolar: 1 Homogenous: 2 Fine speckled/Homogenous: 1
Aquaporin-4 antibody Negative	n= 216 (87%)	32.3 ± 13.9 (Age range: 2-73)	Females: n= 126 (58.3 %) Males: n= 90 (41.6%)	n= 70 (32 %) Females: n= 40 (57.1%) Males: n= 30 (42.8 %)		Fine speckled: 31 Homogenous: 13 Nucleolar: 10 Centrosome: 9 Cytoplasmic Pattern: 7

* The association between anti AQP-4 and ANA positivity was analyzed using the chi-square test, with a p-value < 0.05 considered statistically significant.

In patients positive for AQP-4 antibody, symptoms of transverse myelitis were most common clinical presentation, followed by visual and area postrema symptoms. Different treatment modalities were used in patients diagnosed with NMOSD. All these patients were treated with Intravenous steroids as the first line therapy. Four patients also underwent plasma exchange during hospital stay whereas two patients received intravenous Rituximab. Oral steroids were prescribed to all 32 patients upon discharge with concurrent azathioprine in four patients.

On Follow-up: Patients who had anti-nuclear antibodies in serum were followed for a period of six to twelve months to investigate for development of symptoms associated with autoimmune disorders (Table-III).

**Table-III:** Frequency of symptoms associated with connective tissue/ autoimmune disorders and diagnosis of autoimmune disease on follow up in patients with positive ANA.

Antibodies (n)	Symptoms at 6 months (n)	Diagnosed autoimmune disease at 1 year (n)
AQP-4 and ANA: + *(17)	8	3
AQP-4 ab-, ANA+ ^#^(70)	8	None

*AQP-4 and ANA antibody positive, # AQP-4 antibody negative and ANA positive

At six months follow-up, in NMOSD cohort with positive ANA, five out of 17 patients had complaints of joint pains, involving small joints of hands without any associated swelling and two out of 17 patients had hyper pigmented patches of skin of bilateral lower limbs below knees. On follow up at one year, one of these patients was diagnosed with Systemic Lupus erythematosus by a rheumatologist after having symptoms including pain in small joints of hands, receding hairline and oral ulcers. On laboratory investigations she had positive anti-ds DNA antibodies and positive 2+ Anti-nuclear antibodies and showed a fine speckled pattern of ANA on initial testing. Patient was prescribed Oral steroids and HCQ.

Furthermore, two out of 17 patients were diagnosed with autoimmune thyroid disorders, on one year follow-up by an endocrinologist and were on oral thyroxine. Another patient, with positive anti-AQP4 and ANA gave history of multiple miscarriages. This patient was however lost to follow-up. Rest nine patients from AQP-4 and ANA positive group denied any history of symptoms related to any connective tissue or organ specific autoimmune disorder. In AQP-4 antibody negative and ANA positive group, eight patients had complaints of joint pains on initial inquiry however none of the patients was diagnosed with autoimmune disease at six and 12 months follow up.

## DISCUSSION

According to published literature, association of Aquaporin four antibodies with other autoantibodies has been reported. However, there are few studies exploring this association. We conducted this study to observe frequency and significance of Anti-nuclear antibodies in patients with positive anti-Aquaporin-4 antibody in local cohort.

In our study, Aquaporin-4 antibody positivity was 12.9% which is higher compared to a study done in local Pakistani population by Arshad et al showing AQP-4 positivity to be 2.4%.^6^ Mean age in our patients was comparable to that reported in a previous study done in local NMOSD patients by Haris et al. and in Iranian and Egyptian populations.^7^-^9^ Out of total 32 Aquaporin-4 antibody positive patients, 87.5% were females with 1:7 male to female ratio. This finding is in coherence with other reported data.^10^,^11^ However, it is considerably higher than female: male ratio of 1.25:1 described by Arshad et al. This could be due to low Aquaporin-4 antibody positivity in their study. In our AQP-4 antibody positive cohort, 53% patients had symptoms of transverse myelitis at presentation followed by symptoms of optic neuritis, similar to the observations made in other local and international studies.^6^,^12^ Data related to association of other autoantibodies particularly Anti-nuclear antibodies and autoimmune diseases in NMOSD patient can be found in literature, although scarce. Tatjana et al conducted a study on a cohort of diagnosed NMOSD patients from a Serbian Registry and concluded that AQP4-IgG seropositive NMOSD patients had 5.2-fold higher risk of comorbid autoimmune diseases.^13^

The most frequently reported diseases were autoimmune thyroid disease (15.4%) and Antinuclear antibodies were detected in 54.3% of NMOSD patients. These findings are similar to ANA positivity of 53% in our AQP-4 antibody positive group with two patients diagnosed with autoimmune thyroid disorders at one year follow up. Another study conducted by Theodora et al in European NMOSD patients showed ANAs were detected in 19/23 (82.6%) AQP-4 antibody positive samples.^14^ Furthermore, In the AQP4-IgG seropositive cohort, 13 patients (35.1%) reported autoimmune comorbidities and the most common disorders were SLE followed by autoimmune thyroiditis. A literature review done by Sareh et al also depicted that patients with NMOSD had an increased risk to develop a concurrent autoimmune disease, most frequently Sjogren’s Syndrome followed by Systemic Lupus. Moreover, myasthenia gravis in neurological and autoimmune thyroid diseases in non-neurological organ-specific autoimmune diseases were the most reported comorbidities associated with NMOSD^15^, in this review. However, none of our patient had developed symptoms of Sjogren syndrome or Myasthenia gravis on follow-up, so far.

Contrary to these observations, Pareira et al. reported ANA positivity of 27.3% in their cohort of NMOSD patients and the results of their study underscored that the NMO patients present high frequency of autoantibodies against cellular antigens along with the presence of autoimmune disorders.^16^ While comparing clinical outcomes in NMOSD patients between ANA positive and negative groups, there is published data showing that presence of Antinuclear antibodies is associated with more severe disease activity in NMOSD patients.^5^

### Limitations:

It include short duration of follow-up. Following patients with positive ANA for extended period to see for development of symptoms related to autoimmune diseases would lead to better understanding of association of ANA and autoimmune comorbidities with NMOSD. We also only considered patients who had positive Anti Aquaporin-4 antibodies as NMOSD in this study and could have missed some Aquaporin 4 antibody negative NMOSD patients.

## CONCLUSION

In our study, it has been demonstrated that there is significant association of anti-nuclear antibodies with Anti Aquaporin-4 antibody and NMOSD patients with positive ANA have a higher frequency of developing other autoimmune disorders. Our data also suggests with plausible evidence that physicians should look for other autoimmune comorbidities in patients who have positive Anti Aquaporin-4 antibodies, and these patients should be followed up to look for development of any such disease.

### Authors Contribution:

**FJ:** conception, design, acquisition, analysis, and interpretation of data. Drafted the manuscript.

**SS:** Analysis, interpretation of data and drafted the manuscript.

**AA and TAA:** Conception and design of the study; supervision; interpretation of data; critical review of manuscript for important intellectual content.

All authors have approved the final version of the manuscript and agree to be accountable for all aspects of the work.
